# Chondroitin / Dermatan Sulfate Modification Enzymes in Zebrafish Development

**DOI:** 10.1371/journal.pone.0121957

**Published:** 2015-03-20

**Authors:** Judith Habicher, Tatjana Haitina, Inger Eriksson, Katarina Holmborn, Tabea Dierker, Per E. Ahlberg, Johan Ledin

**Affiliations:** 1 Department of Organismal Biology, Science for Life Laboratory, Uppsala University, Uppsala, Sweden; 2 Department of Medical Biochemistry and Microbiology, Science for Life Laboratory, Uppsala University, Uppsala, Sweden; Columbia University, UNITED STATES

## Abstract

Chondroitin/dermatan sulfate (CS/DS) proteoglycans consist of unbranched sulfated polysaccharide chains of repeating GalNAc-GlcA/IdoA disaccharide units, attached to serine residues on specific proteins. The CS/DS proteoglycans are abundant in the extracellular matrix where they have essential functions in tissue development and homeostasis. In this report a phylogenetic analysis of vertebrate genes coding for the enzymes that modify CS/DS is presented. We identify single orthologous genes in the zebrafish genome for the sulfotransferases *chst7*, *chst11*, *chst13*, *chst14*, *chst15* and *ust* and the epimerase *dse*. In contrast, two copies were found for mammalian sulfotransferases *CHST3* and *CHST12* and the epimerase *DSEL*, named *chst3a* and *chst3b*, *chst12a* and *chst12b*, *dsela* and *dselb*, respectively. Expression of CS/DS modification enzymes is spatially and temporally regulated with a large variation between different genes. We found that CS/DS 4-O-sulfotransferases and 6-O-sulfotransferases as well as CS/DS epimerases show a strong and partly overlapping expression, whereas the expression is restricted for enzymes with ability to synthesize di-sulfated disaccharides. A structural analysis further showed that CS/DS sulfation increases during embryonic development mainly due to synthesis of 4-O-sulfated GalNAc while the proportion of 6-O-sulfated GalNAc increases in later developmental stages. Di-sulfated GalNAc synthesized by Chst15 and 2-O-sulfated GlcA/IdoA synthesized by Ust are rare, in accordance with the restricted expression of these enzymes. We also compared CS/DS composition with that of heparan sulfate (HS). Notably, CS/DS biosynthesis in early zebrafish development is more dynamic than HS biosynthesis. Furthermore, HS contains disaccharides with more than one sulfate group, which are virtually absent in CS/DS.

## Introduction

**Fig 1:**
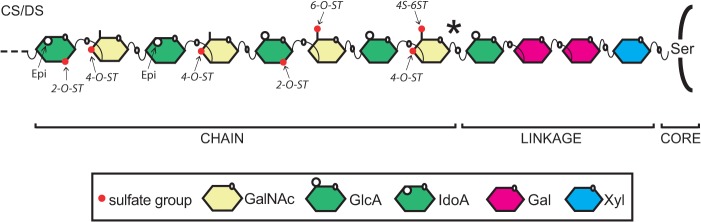
Modification reactions in CS/DS biosynthesis. The CS/DS chain is modified by sulfotransferases and epimerases. A GlcNAc added to the linkage region (asterisk) commits the nascent chain to HS synthesis instead of CS/DS synthesis. Epi: GlcA-C5-epimerase, Gal: galactose, GalNAc: *N*-Acetylgalcotamine, GlcA: glucuronic acid, GlcNAc: *N*-Acetylglucosamine, IdoA: iduronic acid, Ser: Serine, Xyl: xylose, 2-O-ST: 2-*O*-sulfotransferase, 4-O-ST: 4-*O*-sulfotranferases, 4S-6ST: *N*-Acetylgalactosamine-4-sulfate-6-*O*-sulfotransferase, 6-O-ST: 6-*O*-sulfotransferases.

Chondroitin/dermatan sulfate (CS/DS) proteoglycans consist of core proteins with attached linear sulfated polysaccharides of repeating glucuronic acid and *N*-acetylgalacotsamine (GlcA-GalNAc) in CS or iduronic acid and *N*-acetylgalactosamine (IdoA-GalNAc) in DS (Fig. 1). CS/DS is produced by virtually all vertebrate cells and has multiple functions during animal development, including fundamental processes as cell division and cytokinesis [1–3]. CS/DS is deposited extensively in cartilage ECM, where the high overall charge provides electrostatic forces to promote high water content, enabling joints to withstand mechanical compression. CS/DS biosynthesis produces chains varying in length, charge density and sulfation pattern, which affect physical docking to ECM molecules, such as growth factors, thus functioning as an extra level of regulation to dictate the function of CS/DS binding proteins [4, 5].

The biosynthesis of CS/DS is initiated with the synthesis of a GlcA–Gal–Gal–Xyl–O-Ser tetrasaccharide structure attached to one of more than 30 known CS/DS core proteins [5, 6]. This structure constitutes the linkage region for CS/DS as well as for heparan sulfate (HS). In HS biosynthesis, a GlcNAc is added to the linkage region as the fifth sugar followed by repeating additions of GlcNAc and GlcA. In biosynthesis of CS/DS a GalNAc is added to the linkage region (Fig. 1). Sometimes there may be a competition for link structures by CS/DS and HS specific enzymes [7]. However, if HS or CS will be synthesized also depends on other factors such as amino acid properties in close vicinity to the attachment serine residue [8] and sulfation of the link structure [9]. Elongation of CS/DS by repeating additions of GalNAc and GlcA is the result of a concerted action of CS/DS glycosyltransferases [5, 6, 10].

The common sulfate donor, 3′-phosphoadenosine 5′-phosphosulfate (PAPS) is used by all sulfotransferases during CS/DS modification. CS modifications are initiated by the addition of a sulfate group to the GalNAc C-4 or C-6 position by chondroitin 4-*O*- or 6-*O*-sulfotransferases, respectively (Fig. 1). A portion of GlcA residues are converted by DS epimerases to IdoA, and IdoA-GalNAc disaccharides may be sulfated on the C-4 position of the GalNAc residue by a dermatan 4-*O*-sulfotransferase. Subsequently, a subset of 4-*O*-sulfated or 6-*O*-sulfated disaccharides may be disulfated by the sulfation of the C-2 position on GlcA/IdoA by a 2-*O*-sulfotransferase or sulfated at the C-6 position of a previously C-4 sulfated GalNAc residue by a GalNAc4S-6-*O*-sulfotransferase. Typically, the resulting molecule consists of both GlcA and IdoA residues and is hence a hybrid between CS and DS.

Eight CS/DS sulfotransferases were identified in mammals: two chondroitin 6-*O*-sulfotransferases *CHST3* (*C6ST-1*) [11] and *CHST7* (*C6ST-2*) [12], three chondroitin 4-*O*-sulfotransferases *CHST11* (*C4ST-1*) [13], *CHST12* (*C4ST-2*) [13], *CHST13* (*C4ST-3*) [14], one dermatan 4-*O*-sulfotransferase *CHST14* (*D4ST-1*) [15], one *N*-acetylgalactosamine 4-sulfate 6-*O*-sulfotransferase *CHST15* (*GALNAC4S-6ST*) [16] and one uronyl-2-sulfotransferase (*UST*) [17]. Conversion of GlcA residues into the stereoisomer IdoA is mediated by two dermatan sulfate epimerases, *DSE* (*DS-epi1*) [18] and *DSEL* (*DS-epi2*) [19].

A number of genetic alterations in genes involved in the CS/DS biosynthesis have been investigated in human patients [20] transgenic mice [21]. Human patients with mutations in the chondroitin 6-*O*-sulfotransferase *CHST3* gene develop chondrodysplasia and are diagnosed with Larsen syndrome [22, 23], although targeted mutations in the *Chst3* gene in mice did not result in any apparent phenotype [24]. Different homozygous mutations in human *CHST14* and *DSE* have been linked to Ehlers-Danlos syndrome (EDS), where patients display, among other symptoms, distinctive craniofacial dysmorphism [25, 26]. Mice lacking a functional *Chst14* or *Dse* gene have a reduced body size and show increased skin fragility, corresponding to human symptoms [25, 27]. A lower survival frequency of these mice has also been seen. Interestingly, a genome-wide association study suggested CHST11 as a plausible candidate gene for increased osteoarthritis susceptibility [28]. Mice lacking functional *Chst11 (C4ST-1)* develop chondrodysplasia [5, 29] while mice deficient of functional *Chst15 (GalNac4S-6ST)* lack GalNAc 4,6 disulfate residues but develop no apparent abnormalities [30].

In the developing mouse embryo *chst11* is expressed in the branchial arches and the limb buds as well as in the notochord [31]. *Chst12 (C4ST-2)* is expressed in the stomach and gut as well as in the skeleton in a 14,5 day old mouse embryo [32]. *Chst13 (C4ST-3)* is expressed in the liver and kidney, in the bones of the upper and lower jaws and in the eye socket [33]. No expression was found in the developing brain, in contrast to adult brain *Chst13* expression. *Chst15* is expressed during mouse embryonic development at E8.5 in the neuroepithelium of the forebrain region, and is particularly strong in the dorsal aspects of the neural folds [22]. Furthermore, expression was detected in the pharyngeal region and the foregut as well as in the somites [22].

Little is known of the spatio-temporal regulation of CS/DS biosynthesis during zebrafish development. Hayes and coworkers have used antibodies recognizing CS/DS sulfation motifs to study zebrafish skeletogenesis, and describe non-sulfated disaccharides and disaccharides with sulfate groups at C-4 and C-6 positions (Fig. 1), but also more subtle changes in sulfation pattern indicating dynamic control of CS/DS biosynthesis during development [34]. Expression of CS/DS modification enzymes during zebrafish embryonic development has not been systematically studied but a study of zebrafish c*hst11* (*C4ST-1*) shows persistent expression in somites and brain, and transient expression in the pectoral fins [35].

We have previously characterized CS/DS polymerization during zebrafish development [10] and in this study we systematically investigate the phylogeny and expression of all known CS/DS modification enzymes as well as the temporal variation of CS/DS structures. We conclude that CS/DS biosynthesis is a highly dynamic regulated process during vertebrate development.

## Experimental Procedure

### Ethic statement

All animal experiments in this project were approved by Uppsala Djurförsöksetiska nämnd, Uppsala, Sweden (Permit number C262/11).

### Animals

Wild-type zebrafish (AB, WIK) embryos were obtained in natural crosses and cultured in system water. 0.003% 1-phenyl-2-thiourea (Sigma) was added to the water to inhibit pigmentation. Embryos were anesthetized in tricaine methanesulfonate (Sigma-Aldrich) prior to fixation and staged using hours post fertilization (hpf) [36].

### Phylogenetic analysis

Gene and protein sequences for CHST3, CHST7, CHST11–15, UST, DSE, and DSEL from human, mouse, zebrafish and stickleback were collected from the Ensembl database [37]. Ensembl accession numbers are displayed in Table 1. Amino acid sequences were aligned with Clustal Omega [38]. The default alignment parameters were applied. The sequences were realigned and bootstrapped 1000 times using SEQBOOT from Phylip 3.695v [39]. Protein distances were calculated using PROTDIST. The Jones–Taylor–Thornton matrix was used for the calculation. The neighbor-joining trees were calculated from the 1000 different distance matrixes, previously generated with PROTDIST, using NEIGHBOR from Phylip 3.695. Majority rule consensus tree was constructed with CONSENSE from the same package. The trees were plotted using TreeView 1.6.6 [40].

**Table 1:** Accession numbers of CS/DS modification enzymes.

Human/Mouse	Alternative name	Ensembl Nr ENSG00000	Chr. Pos.	Ensembl Nr ENSMUSG000000	Chr. Pos.	Zebrafish	aa %	Ensembl Nr ENSDARG000000	Chr. Pos.	Stickleback	Ensembl Nr ENSGACG000000	Chr. Pos.
CHST3	Chondroitin 6-O-sulfotransferase 1	122863	10:73.72m	57337	10: 60.18m	chst3a	58	61070	13: 29.74m	chst3a	02438	grV:1.01m
						chst3b	53	77844	12: 50.62m	chst3b	08386	grVI:9.61m
CHST7	Chondroitin 6-sulfotransferase 2	147119	X:46.43m	37347	X: 20.06m	chst7	53	44341	6: 37.57m	chst7	13833	grVIII:17.98m
CHST11	Chondroitin 4-O-sulfotransferase 1	171310	12:104.85m	34612	10: 82.99m	chst11	79	34375	4: 8.24m	chst11	19340	grIV:24.26m
CHST12	Chondroitin 4-O-sulfotransferase 2	136213	7:2.44m	36599	5: 140.51m	chst12a	61	28786	3: 41.72m	chst12a	07992	grXI:4.98m
						chst12b	55	77198	1: 10.92m	chst12b	16733	grIX: 5.13m
						si:dkey-26i13.4		94981	1: 10.82m			
						si:dkey-26i13.3		95482	1: 10.81m			
						si:dkey-26i13.5		93636	1: 10.80m			
						si:dkey-26i13.6		92200	1: 10.79m			
						si:dkey-26i13.7		92242	1: 10.78m			
CHST13	Chondroitin 4-O-sulfotransferase 3	180767	3:126.24m	56643	6: 90.31m	chst13	52	04018	23: 34.99m	chst13a	10830	grXVII:11.00m
										chst13b	04801	grXII:5.08m
CHST14	Dermatan 4-sulfotransferase 1	169105	15:40.76m	74916	2:118.93m	chst14	59	43011	20: 25.78m	chst14	14327	grVIII:18.79m
CHST15	N-acetylgalactosamine 4-S 6-O-sulfotransferase	182022	10:125.77m	30930	7:132.24m	chst15	56	74527	17: 21.34m	chst15a	02542	grV:1.18m
										chst15b	09700	grVI:11.74m
UST	DS/CS 2-O ulfotransferase	111962	6:149.07m	47712	10:8.20m	ust	67	06044	20:31.45m	ust	11589	grXVIII:11.86m
DSE	Chondroitin-glucuronate 5-epimerase	111817	6:116.58m	39497	10:34.15m	dse	66	17988	20:0.55m	dse	06171	grXVIII:3.37m
DSEL		171451	18:65.17m	38702	1:111.86m	dsela	62	74504	24:0.05m	dsel	04870	grXXI:10.44m
						dselb	58	61461	2:27.46m			

CS/DS modification enzymes and their alternative names in human, mouse, zebrafish and stickleback. Amino acid (aa) sequence identity (%) to human orthologues is displayed for predicted zebrafish proteins. Ensembl accession numbers and chromosomal positions are also presented.

### cDNA cloning

All probes were amplified by PCR from zebrafish cDNA template, transcribed using T7 polymerase and DIG-labeling kit (Roche). Nucleotide removal was performed with the RNeasy kit (Qiagen) and the riboprobes were checked on an agarose gel. Primers for the respective genes are listed in Table 2.

**Table 2:** Primers used to make RNA probes for the in situ hybridization of CS/DS modification enzymes.

Gene	Forward Primer	Reverse Primer
*chst3a*	TGTGGGCGAGTTCTTTAAC	AAGTTTCATGGTCGGTCCAC
*chst3b*	TGTGGCTTTGGTCATCATAGA	GATGAAGCGCATGTGCAG
*chst7*	ATGAAGAGAAGGCTTCAGAAAAAATACATCATCTTA	TTAGGTCTTCTCCATGTTCCTCTTCTGATAATCCAG
*chst11*	TGGAGATCCCTTCCAATGAG	CAGGGCTTCAGACCTCAGTC
*chst12a*	TCCTGGAGACCCTCACAATC	TTTGTCTCCAGTGCTCGTTG
*chst12b*	GCAGCTGCAGTTTTTCCTTC	ATGCTCATTGAACGGCTTCT
*chst13*	AAACAGAAGCGGTTTGTTCTCCG	TCATCTCAACTTCAAGTACGCAGGCAT
*chst14*	TTTCAAGAAAGCGCAGAAATCAGAGGTT	TAGTGCCTGCAGTATTCAGTGGTTGTGT
*chst15*	CCACAAGTATGGCCTCCTAAGCT	CACAGAAAAGCAGGGTCCTTTAA
*dse*	TCAGCGTACATGGAGACTCG	GCTGTTAGCATTGTGCCTGA
*dsela*	TGTTTGACTTTGGGGTCACA	CAGGAAGGGCAGCTTTAGTG
*dselb*	AGCTGCCGAATTCAAAAAGA	CTCTGGACCACTCACAAGCA

### Whole mount in situ hybridization

In situ hybridization was performed essentially as previously described [41]. Zebrafish embryos and larvae of different stages were fixed with 4% paraformaldehyde in phosphate buffered saline (PBS), dehydrated in methanol and stored at-20°C. Upon using, embryos were rehydrated and permeabilized with Proteinase K (5μg/ml). Hybridization was performed at 70°C and riboprobes were detected with BM purple AP Substrate precipitating solution (Roche). Embryos and larvae were cleared in glycerol and photographed with a Nikon SMZ1500 Stereomicroscope. Images and figures were adapted in Adobe Photoshop and Adobe Illustrator.

### Structural Analysis of CS/DS and HS by Reverse Phase Ion Pair (RPIP)-HPLC

CS/DS and HS were isolated from zebrafish embryos and larvae at different stages in duplicate or triplicate samples with 250 embryos at 30 hpf and 100 embryos at 2 days post fertilization (dpf), 3 dpf and 4 dpf. CS/DS was degraded into disaccharides by enzymatic cleavage, and detected in a HPLC-based system essentially following the protocol as described earlier [42], with the modification of washing the DEAE column with 0.4M NaCl to remove hyaluronic acid. In short, GAGs were isolated by proteolytic cleavage, nuclease treatment, and DEAE ion exchange chromatography. The purified GAGs were cleaved with chondroitinase ABC and 10% of the sample was used to identify CS/DS disaccharide components. HS was purified from cleaved CS/DS disaccharides by a second DEAE purifications step followed by cleavage with heparin lysase I-III. The two generated samples of CS/DS and HS disaccharides were subjected to RPIP-HPLC analysis followed by post-column derivatization with cyanoacetamide and detection in a fluorescence detector, essentially as described [42]. The identity and the amount of the disaccharides were established by comparing the samples with CS/DS and HS disaccharide standards, respectively.

## Results

### Most CS/DS modification enzymes are present as single orthologues in zebrafish

**Fig 2:**
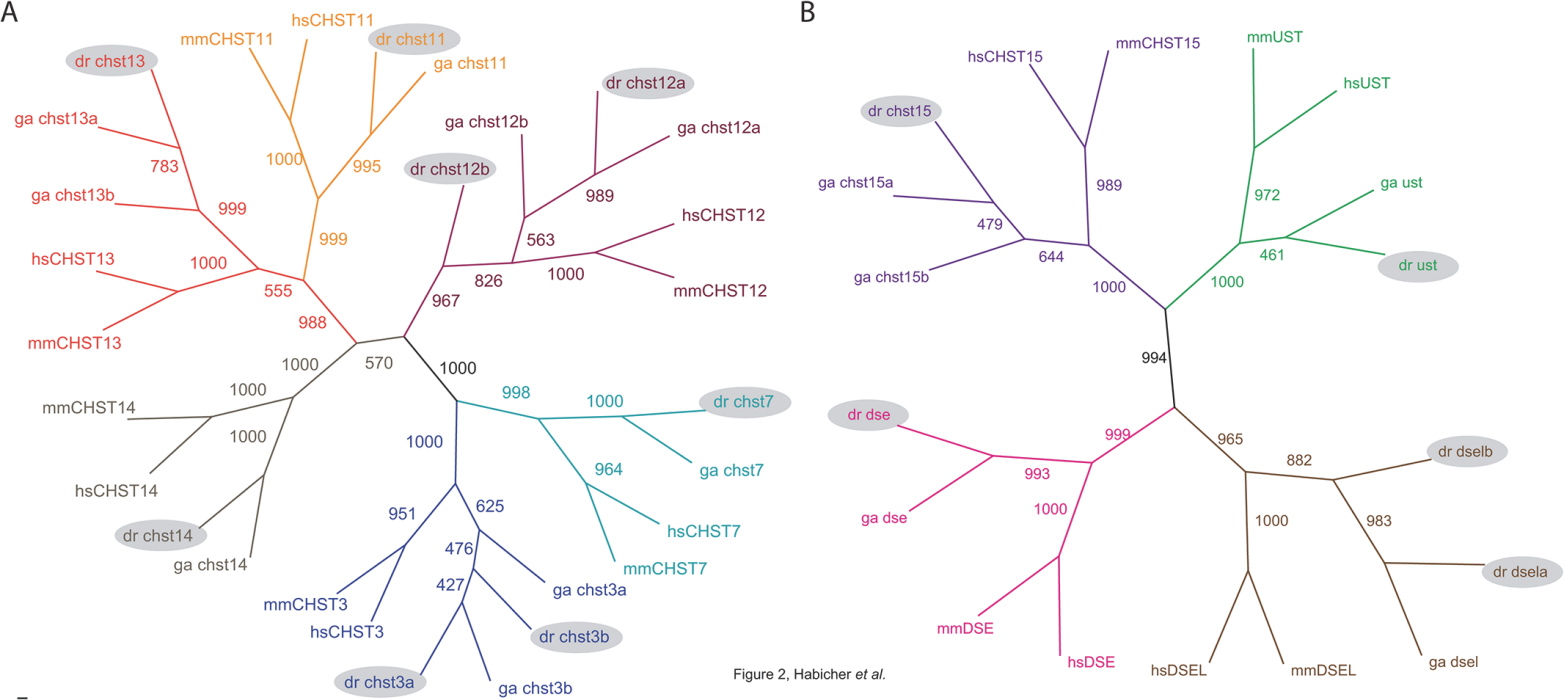
Phylogenetic analyses of CS/DS 4-*O*- and 6-*O*-sulfotransferases (A) and other CS/DS modification enzymes (B). The consensus neighbor-joining trees were calculated with 1000 bootsrap replicates. Zebrafish orthologues are marked with grey shadow. Species names are abbreviated as hs, human; mm, mouse; dr, zebrafish, and ga, stickleback. The accession numbers are listed in the Table 1.

BLAST searches using previously reported amino acid sequences of human and mouse CS/DS modification enzymes [5, 43] as templates were performed to identify orthologous genes in zebrafish (*D*. *rerio*). Amino acid sequence alignments of identified zebrafish enzymes and human orthologues can be found in S1 Fig. We included another teleost fish, stickleback (*G*. *aculeatus*), into the analysis in order to better understand the divergence of genes in different teleost lineages after the teleost specific genome duplication. One orthologue of CS/DS 6-*O*-sulfotransferase *CHST7* and two orthologues of CS/DS 6-*O*-sulfotransferases *CHST3*, named *chst3a* and *chst3b*, were identified in both teleost species, sharing 53–58% identity with human proteins (Fig. 2A). Three genes in the group of CS/DS 4-*O*-sulfotransferases *CHST11*, *CHST13* and *CHST14* displayed single orthology to zebrafish genes *chst11*, *chst13* and *chst14*, respectively. However, in stickleback two co-orthologues of *CHST13*, *chst13a* and *chst13b* were identified. Zebrafish 4-*O*-sulfotransferases share 52–79% amino acid identity with corresponding human proteins.

Notably, seven sequences show orthology to the human CS/DS 4-*O*-sulfotransferase *CHST12*. Zebrafish *chst12a* displays 61% identity in protein sequence and is located on chromosome 3, while *chst12b* shares 55% identity with human protein and is located on chromosome 1. The additional five genes are located as tandem copies in close proximity to *chst12b* on chromosome 1 (Table 1). There is evidence for expression of *chst12a* and *chst12b* in zebrafish in the form of pooled RNASEQ data, displayed in the Ensembl database, but no conclusive evidence that the tandem duplicates of *chst12*, named *chst12 (3-*, *4-*, *5-*, *6-* and *7)*, are functional. In stickleback we find two copies of *chst12*, where one is a clear orthologue of zebrafish *chst12a*. With the help of additional analysis of *chst12* genes in other teleosts, we could see a variation in the number of *chst12* copies between different teleost species (data not shown). There is only one single *chst12* gene in gar, a group of fish that diverged prior to the teleost specific genome duplication, indicating that the rest of the genes in teleosts are lineage specific duplicates.

The human 4-sulfate 6-*O*-sulfotransferase *CHST15* does not belong to the same family as CS/DS 6-*O*-sulfotransferases. There is one orthologue of human 4-sulfate 6-*O*-sulfotransferase *CHST15* in zebrafish, named *chst15* sharing 56% identical amino acid residues. Other teleosts like stickleback (Fig. 2B), cod, fugu, medaka and platyfish have two co-orthologues of this sulfotransferase gene, named *chst15a* and *chst15b* (data not shown). A single orthologue was identified for CS/DS 2-*O*-sulfotransferase (ust) in teleosts, where zebrafish *ust* is 67% identical to the human protein sequence. There are two dermatan sulfate epimerase genes in mammals, *DSE* and *DSEL*. Human *DSE* shares 66% amino acid identity with the zebrafish orthologue *dse*. In the zebrafish genome *dsel* seems to be duplicated. *dsela* displays higher identity of the predicted amino acid sequence to the human DSEL (62%) and stickleback dsel (73%) compared to *dselb* (58% and 63%) (Table 1).

### CS/DS modification enzymes are spatially and temporally regulated in early zebrafish development

#### Chondroitin and Dermatan Sulfate 4-O-sulfotransferases

**Fig 3:**
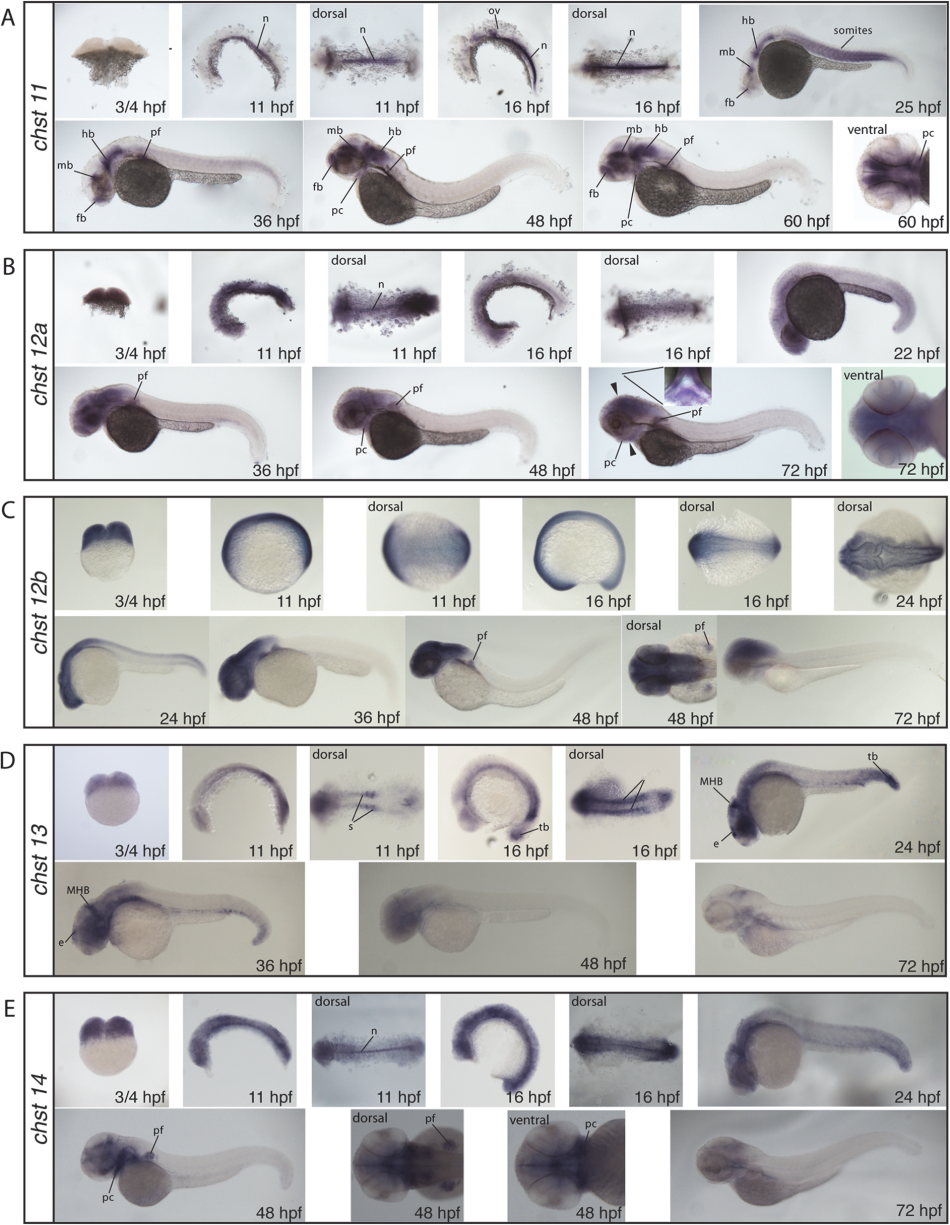
Whole mount in situ hybridization of the CS/DS 4-*O*-sulfotransferases *chst11* (A), *chst12a* (B), *chst12b* (C), *chst13* (D) and *chst14* (E). All images, if not otherwise stated in the figure, are shown in lateral views. Arrowheads indicate the position of the transverse section shown in B at 72 hpf. e: epiphysis, hb, hindbrain, mb: midbrain, MHB: midbrain-hindbrain boundary, n: notochord, ov: otic vesicle, pc: pharyngeal cartilage, pf: pectoral fin, s: somites, tb: tailbud

With the exception of c*hst11*, all 4-*O*-sulfotransferases show expression at the 2-cell stage (¾ hours post fertilization (hpf)) indicating maternal contribution (Fig. 3). We find that *chst11*, *chst12a* and *chst14* are expressed in the notochord at the somite stages (11–16 hpf) (Fig. 3), in accordance with previously published findings for *chst11* [35]. Staining of *chst11* is shifted from the notochord to the somites, the hindbrain, the midbrain and weak staining is seen in the forebrain at 24 hpf (Fig. 3A). By 36 hpf, the somite expression fades; the forebrain, some cells of the midbrain and the pectoral fin bud show staining, and the hindbrain has a very strong expression (Fig. 3A). At 48 and 60 hpf, the for-, mid- and hindbrain as well as the pectoral fins remain stained and in addition, forming pharyngeal cartilages weakly express *chst11* (Fig. 3A). *chst12a* shows a weak overall expression with stronger expression in the anterior regions at 24 hpf. At 36 hpf, the head and pectoral fins are prominently stained (Fig. 3B). From 48 hpf, *chst12a* expression is localized to the head, the pectoral fins and the pharyngeal cartilage (Fig. 3B). *chst12b* shows at the 5- and 15- somite stages (11–16 hpf) weak overall expression (Fig. 3C). At 24 hpf, expression becomes stronger in the central nervous system, and at 36 and 48 hpf, the central nervous system and pectoral fins are prominently stained. At 72 hpf, this staining is slightly weaker (Fig. 3C). c*hst13* displays clear labeling in the somites and the tail bud at 5- and 15-somite stages (11–16 hpf) (Fig. 3D). At 24 hpf, expression in the tail bud, the midbrain-hindbrain boundary (MHB) and a region overlapping with the epiphysis stains (Fig. 3D). By 36 hpf, staining of *chst13* is still prominent in the midbrain-hindbrain boundary and the epiphysis, and at 48 and 72 hpf, staining is weak and restricted mainly to the head (Fig. 3D). At 24 hpf, *chst14* displays strong labeling in the head region and is prominent in the MHB (Fig. 3E). At 48 hpf, strong staining is detected in the pharyngeal cartilage and in the pectoral fins (Fig. 3E).

#### Chondroitin Sulfate 6-O-sulfotransferases

**Fig 4:**
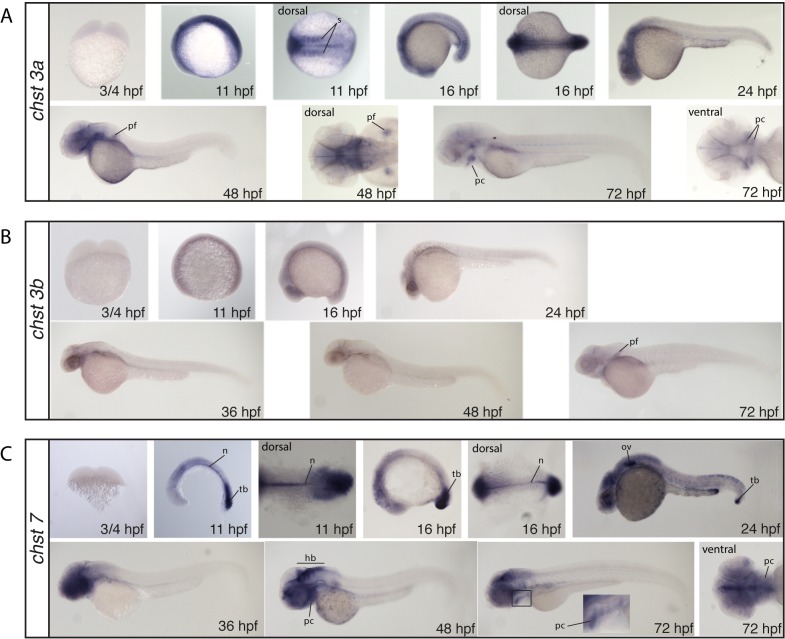
Whole mount in situ hybridization of the CS/DS 6-*O*-sulfotransferases *chst3a* (A), *chst3b* (B) and *chst7* (C). All images show lateral views, if not otherwise stated in the figure. hb: hindbrain, n: notochord, ov: otic vesicle, pc: pharyngeal cartilage, pf: pectoral fin, tb: tailbud, s: somites

c*hst3a*, *chst3b* and *chst7* lack staining at the 2-cell stage (¾ hpf) indicating no maternally deposited mRNA (Fig. 4). The c*hst3a* probe shows weak overall staining at the 5- and 15-somite stages (11–16 hpf), as well as strong somite staining (Fig. 4A). At 24 and 48 hpf staining is limited to the head and the pectoral fins (at 48 hpf). At 72 hpf, staining is restricted to the pharyngeal cartilages and the pectoral fins (Fig. 4A). *chst3b* displays only very weak staining from the 5 somite stage (11 hpf) to 48 hpf (Fig. 4B). At 72 hpf, the pectoral fins show increased staining (Fig. 4B). At the 5- and 15-somite stage (11–16 hpf), the *chst7* probe strongly stains the notochord and tail bud (Fig. 4C). At 24 hpf, staining is still detected at the tail bud but starts shifting to the head region where it gets prominent from 36 hpf (Fig. 4C). At 48 and 72 hpf the hindbrain stains strongly as well as the pharyngeal cartilages. (Fig. 4C).

**Fig 5:**
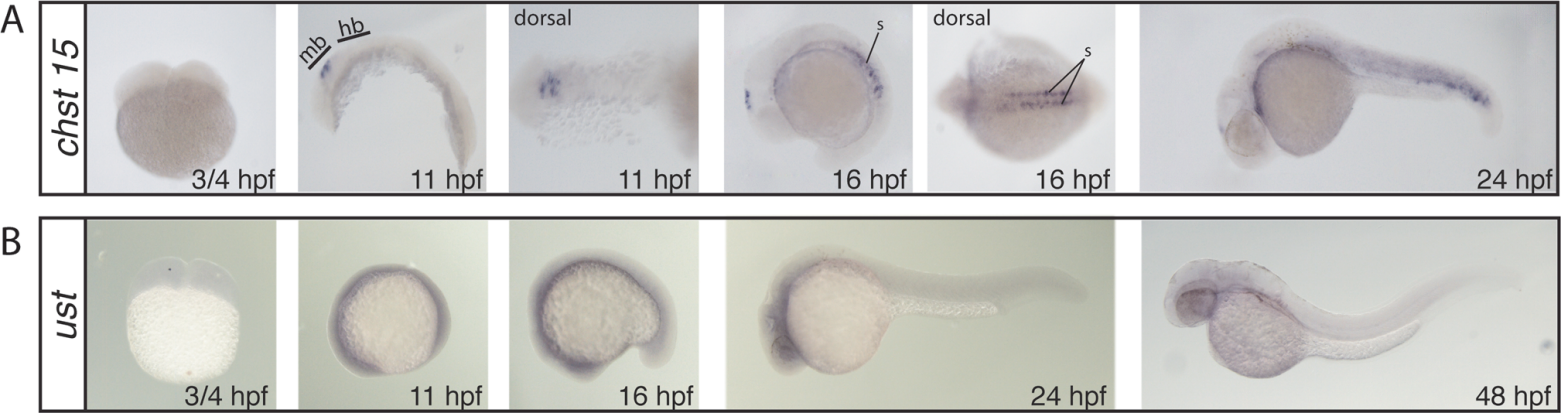
Whole mount in situ hybridization of *N*-acetylgalactosamine 4-sulfate 6-*O*-sulfotransferase, *chst15* (A) and CS/DS 2-*O*-sulfotransferase, *ust* (B). All images show lateral views, if not otherwise stated in the figure. mb: midbrain, hb: hindbrain, s: somites

*N-Acetylgalactosamine 4-sulfate 6-O-sulfotransferase and uronyl-2-sulfotransferase chst15* and *ust* show no staining at the 2-cell stage (¾ hpf) indicating no maternal contribution (Fig. 5). At the 5-somite stage (11 hpf) *chst15* displays a very distinct staining in a subset of cells in the dorsal midbrain (Fig. 5A). This staining persists at the 15-somite stage (16 hpf), where in addition somites stain strongly (Fig. 5A). At 24 hpf, weak somite staining is still present but fades after this stage (Fig. 5A). The probe for *ust* shows weak overall staining at 5- and 15-somite stages (Fig. 5B). At 24 hpf, weak staining in the head region is observed, while at 48 hpf almost no staining is detected (Fig. 5B).

#### Dermatan sulfate epimerases

**Fig 6:**
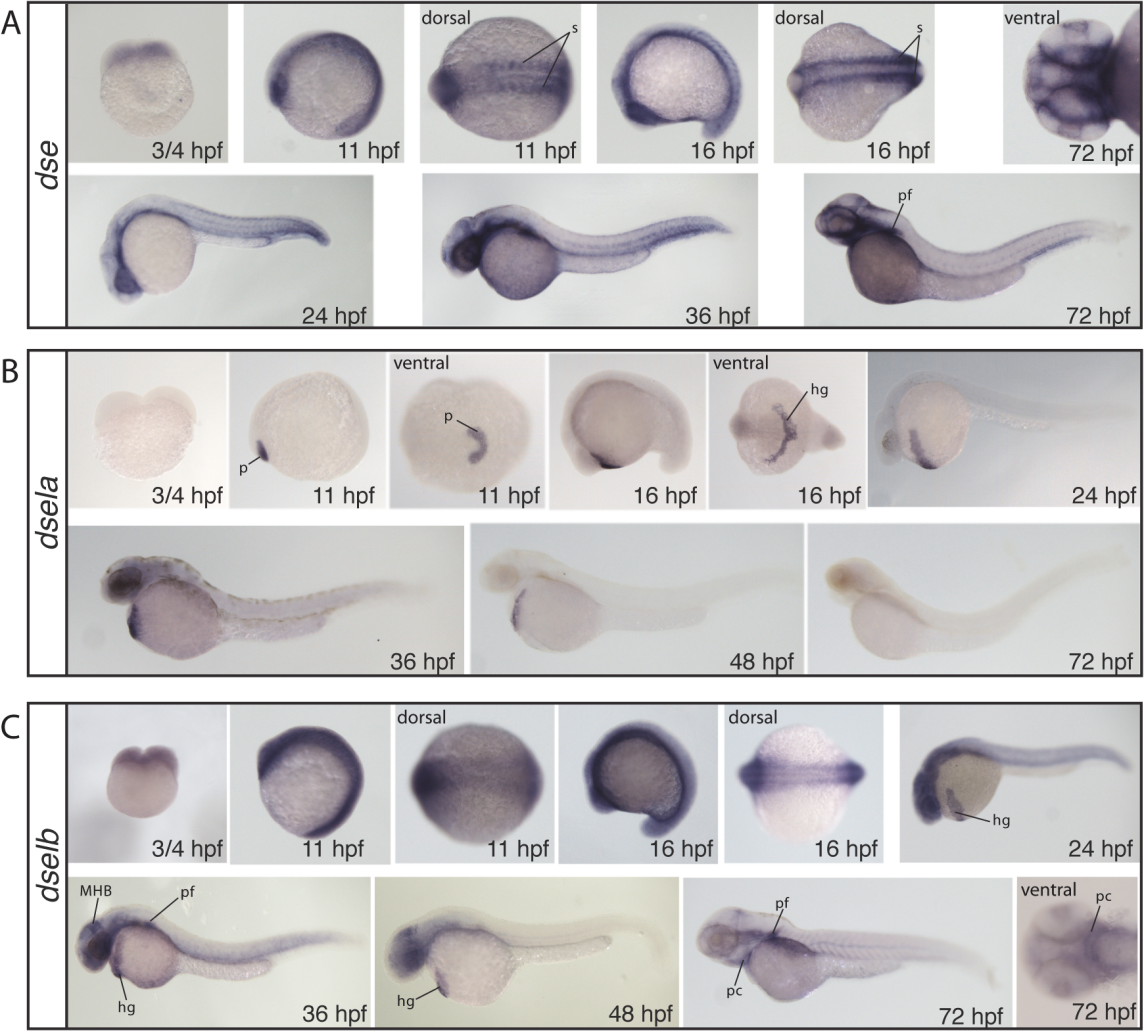
Whole mount in situ hybridization of the DS epimerases *dse* (A), *dsela* (B) and *dselb* (C). All images show lateral views, if not otherwise stated in the figure. hg: hatching gland, MBH: midbrain-hindbrain boundary, p: polster, pc: pharyngeal cartilage, pf: pectoral fin, s: somites

Both probes for *dse* and *dselb* stain at the 2-cell stage (¾ hpf), indicating maternal deposition of mRNA (Fig. 6A,C) while no staining was seen at this stage for *dsela* (Fig. 6B). A general weak staining of *dse* is detected at the 5- and 15-somite stage (11–16 hpf) while somites and the eyes stain strongly (Fig. 6A). At 24 and 36 hpf, only the posterior end of the somites remain stained and staining appears strong in the most ventral regions of the head (Fig. 6A). At 72 hpf staining is still strong and general in the most ventral regions of the head and pectoral fins (Fig. 6A). *dsela* stains very strong in the polster at the 5-somite stage and shows clear staining in cells of the hatching gland at the 15-somite stage (Fig. 6B). The expression in the hatching gland remains persistent at 24, 36 and 48 hpf, while no staining is detected at 72 hpf (Fig. 6B). *dselb* shows a general expression at the somite stages, and at 24 hpf, somites as well as the head region and the hatching gland are stained (Fig. 6C). At 36 and 48 hpf, in addition to the hatching gland, also the midbrain-hindbrain boundary and the pectoral fins are strongly labeled (Fig. 6C). By 72 hpf, staining is weak and restricted to the pectoral fins and the cartilage elements (Fig. 6C).

#### Disulfated disaccharides are rare in zebrafish CS/DS

**Fig 7:**
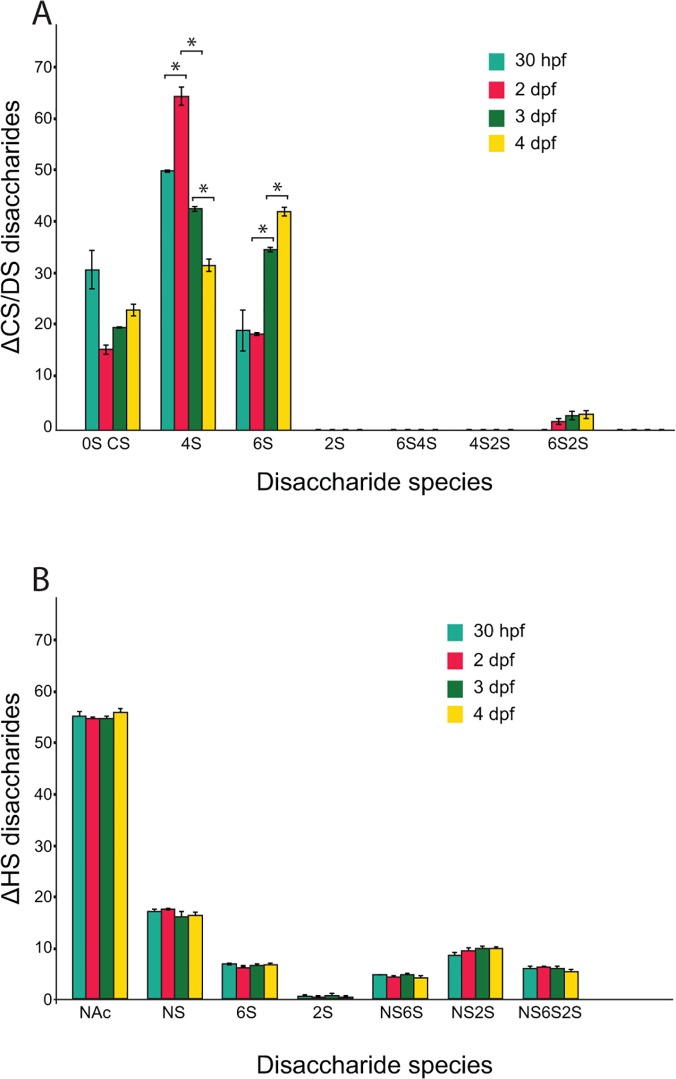
Disaccharide composition of CS/DS (A) and HS (B) as measured by RPIP-HPLC analysis. **Significant differences (p-value <0.01) in the proportion of specific CS/DS disaccharide species are indicated (star) (A).** In contrast, the changes in proportion of specific HS disaccharide species during development between 30 hpf to 4 dpf are not significant (p-value >0.01). (B). Disaccharide species for CS/DS are indicated as 0S CS (ΔHexA- GalNAc/ΔHexA-GlcNAc), 4S (ΔHexA-GalNAc4S), 6S (ΔHexA-GalNAc6S), 2S (ΔHexA2S-GalNAc), 6S4S (ΔHexA-GlcNAc4S6S), 4S2S (ΔHexA2S-GalNAc4S), and 6S2S (ΔHexA2S-GalNAc6S). Disaccharide species for HS are indicated as NAc (ΔHexA-GlcNAc), NS (ΔHexA- GlcNS), 6S (ΔHexA-GlcNAc6S), 2S (ΔHexA2S-GlcNAc), NS6S (ΔHexA-GlcNS6S), NS2S (ΔHexA2S-GlcNS), and NS6S2S (ΔHexA2S-GlcNS6S).

We next investigated the relative sulfation of CS/DS during early zebrafish development. CS/DS was isolated from zebrafish embryos at different developmental stages, cleaved into disaccharides and analyzed by reverse-phase ion-pairing chromatography as previously described [42]. We found that CS/DS sulfation increases from 70 sulfate groups per 100 disaccharides at 30 hpf to 80 sulfate groups 2–4 days post fertilization (dpf) (data not shown). 4-*O*-sulfate groups are the most common modification (Fig. 7A), in accordance with the strong expression of CS/DS 4-*O*-sulfotransferases (Fig. 3). The proportion of 6-*O*-sulfated disaccharides increases from 2dpf (Fig. 7A) correlating with a massive increase in CS/DS synthesis in pharyngeal cartilage [10], suggesting that a high proportion of 6-*O*-sulfation marks CS/DS synthesis in the cartilage during early zebrafish development. Notably, only low amounts of 2-*O*-sulfation and disulfated disaccharides could be detected (Fig. 7A), in agreement with the limited spatial expression of the *ust* and *chst15* enzymes (Fig. 5).

We next compared the changes in CS/DS modifications to HS. Interestingly, compared to the changes in CS/DS modifications (Fig. 7A), HS modifications remains stable during zebrafish development (Fig. 7B).

We conclude that CS/DS sulfation during early zebrafish development is a dynamic process dominated by variable proportions of 6-*O*- and 4-*O*-sulfated disaccharides with a marginal contributions of 2-*O*-sulfated and disulfated disaccharides.

## Discussion

In this study we present a characterization of what, to the current understanding of CS/DS biosynthesis [2, 4, 5, 43], is the complete set of zebrafish enzymes that modify CS/DS.

### The phylogenetic analysis

The zebrafish and stickleback lineages diverged early in teleost evolution but after the teleost-specific genome duplication. Subfunctionalization or neofunctionalization of duplicated genes, or gene loss of one of the duplicates, has occurred both before and after the separation of the two lineages [44]. Our phylogenetic analysis of CS/DS modification enzymes identified orthologous genes of all mammalian enzymes in the teleosts zebrafish and stickleback (Fig. 2).

We have analyzed the orthologous relationship between human, mouse, stickleback, and zebrafish genes and found that three mammalian genes, *CHST3*, *CHST12*, and *DSEL* are present as duplicates in zebrafish. In comparison, in the stickleback genome, *dsel* is present as a single orthologue and the zebrafish single orthologues *chst13* and *chst15* are duplicated. Our phylogenetic analysis furthermore indicates that the two families of 4-*O*- and 6-*O*-sulfotransferases are more similar to each other than to the family of 4-sulfate 6-*O*-sulfotransferase. This family is represented by a single gene, *CHST15*, in most vertebrates, including zebrafish, whereas some other teleost fishes such as stickleback, medaka and pufferfishes, retain both copies of the gene.

### CS/DS biosynthesis is dynamic during embryo development

Our analysis of GAG structures, isolated from whole zebrafish embryos, reveals that CS/DS biosynthesis is dynamic during early zebrafish development compared to an apparent overall stability of HS biosynthesis (Fig. 7). This does not exclude changes in HS structures in certain tissues. The variation of modifications in CS/DS structure during early zebrafish development is characterized by an increase in total sulfation with changes in the proportion of 4-*O*-sulfated respective 6-*O*-sulfated disaccharides (Fig. 7A). The shift from 4-*O*-sulfation to 6-*O*-sulfation and the relative absence of di-sulfated disaccharides during zebrafish embryogenesis and early larval development are reminiscent of *Xenopus laevis* development [45]. Evidence has accumulated for interactions between CS/DS and a large number of proteins (reviewed in [2, 4, 5]) and it is an intriguing possibility that CS/DS structure, regulated by combinatorial expression of CS/DS modification enzymes (Fig. 3–6), mediates evolutionary conserved instructive functions during animal development. The ratio 4-*O*-sulfated / 6-*O*-sulfated disaccharides has also been shown to be crucial during mouse neural development [46]. However, it should be noted that HS disaccharides with multiple sulfate groups, believed to be most important for HS-protein interaction [47], are common during zebrafish development (Fig. 7B). In contrast, CS/DS disaccharides with multiple sulfate groups are rare (Fig. 7A). Also the restricted expression of Chst15 and Ust (Fig. 5), known to be necessary for synthesis of di-sulfated CS/DS disaccharides (reviewed in [5]), suggests that CS/DS disaccharides during zebrafish development are usually decorated with single sulfate groups.

### Composition of CS/DS in zebrafish cartilage and bone

CS/DS is accumulated in zebrafish larvae cartilage structures in large amounts [7] and both CS/DS glycosyltransferases [10], 4-O-sulfotransferases (Fig. 3) and 6-*O*-sulfotransferases (Fig. 4) are distinctly expressed in cartilage structures during zebrafish development. This suggests cartilage CS/DS to be dominated by non-sulfated, 4-*O*-sulfated and 6-*O*-sulfated CS/DS disaccharides, also indicated by the whole embryo composition analysis (Fig. 7). These components can further be detected in most cartilage and bone structures by antibodies recognizing CS/DS motifs in zebrafish larvae at 4 dpf and 8 dpf [34] and similar proportions of different CS/DS disaccharides is found in vertebral CS/DS during zebrafish ageing [48].

### Cooperation between biosynthesis enzymes

CS/DS biosynthesis enzymes produce tissue specific structures during zebrafish development [34] presumably as a result of differential expression of CS/DS modification enzymes (Figs. 3–6). However, we have previously shown that it is difficult to predict the *in vivo* effect of varying amounts of biosynthesis enzymes, indicating a complex regulation of CS/DS biosynthesis [7, 49]. Interestingly, evidence has recently accumulated suggesting that cooperation between CS/DS glycosyltransferases and CS/DS modification enzymes strongly affects CS/DS biosynthesis. Expression of CHST11 (C4ST-1) has been shown to be critical for synthesis of long CS/DS chains in mouse fibroblasts [50] and it was further shown that chain elongation by the CS/DS glycosyltransferase CSGALNACT2 depends on CHST11 (C4ST-1) expression [51]. During zebrafish development, such cooperation would be most likely to occur in the notochord and in brain tissue with strong expression of both Csgalnact2 [10] and Chst11 (Fig. 3). In contrast, cooperation between CSGALNACT1 and CHST12 affects CS/DS biosynthesis differently by increasing the number of chains attached to a core protein rather than affecting chain length [52, 53]. While *chst12a* and *chst12b* are widely expressed (Fig. 3), the restricted expression of *csgalnact1* [10] would suggest that such cooperation would be manifested primarily in pharyngeal cartilage structures. Furthermore, the proportion of GlcA to IdoA in CS/DS chains appears to be controlled by functional cooperation between CHST14 (D4ST-1) and DSE (DS-epi1) [54, 55]. *chst14* expression is widespread in zebrafish embryos (Fig. 3) while the strong expression of *dse* in pharyngeal cartilage and the notochord suggests that functional collaboration resulting in an elevated proportion of IdoA between these enzymes may occur in these structures (Fig. 6). As only a few studies so far have investigated the role of enzymatic collaboration during CS/DS modification, more examples might well be revealed in future studies. It should also be noted that the role of CS/DS enzymes with the ability to catalyze the same enzymatic reaction is still poorly investigated in organisms. For example, a confirmation of the role for CHST7 in CS/DS biosynthesis during animal development is still lacking.

### Conclusions and future directions

In this study we present a detailed phylogenetic analysis of CS/DS modification enzymes and we show that CS/DS modification enzymes are differentially expressed while CS/DS structure varies significantly during zebrafish development. This study together with our previous characterization of zebrafish CS/DS glycosyltransferases [10] thus lay the foundation to design experiments in zebrafish strains with genetically manipulated CS/DS biosynthesis to further understand the regulation of CS/DS biosynthesis, the function of CS/DS modification enzymes and the subsequent effects on embryonic development.

## Supporting Information

S1 Fig Amino acid sequence alignment of the CS/DS modification enzymes in human (hs) and zebrafish (dr) constructed with Clustal Omega. **A**, alignment of CS/DS 4-*O* sulfotransferases. **B**, CS/DS 6-*O* sulfotransferases. **C**, CS/DS 4-sulfate 6-*O* sulfotransferases. **D**, CS/DS 2-*O*-sulfotransferases and **E**, CS/DS epimerases. Identical amino acids are marked with red letters on yellow background, conserved residues are indicated by blue letters on light blue background and blocks of similar residues marked with black letters on green background.
(PDF)
