# Supplementary material for: Chondroitin / Dermatan Sulfate Modification Enzymes in Zebrafish Development
Source: PLoS One. 2015 Mar 20;10(3):e0121957. doi: 10.1371/journal.pone.0121957 (PMC4368567; doi:10.1371/journal.pone.0121957)

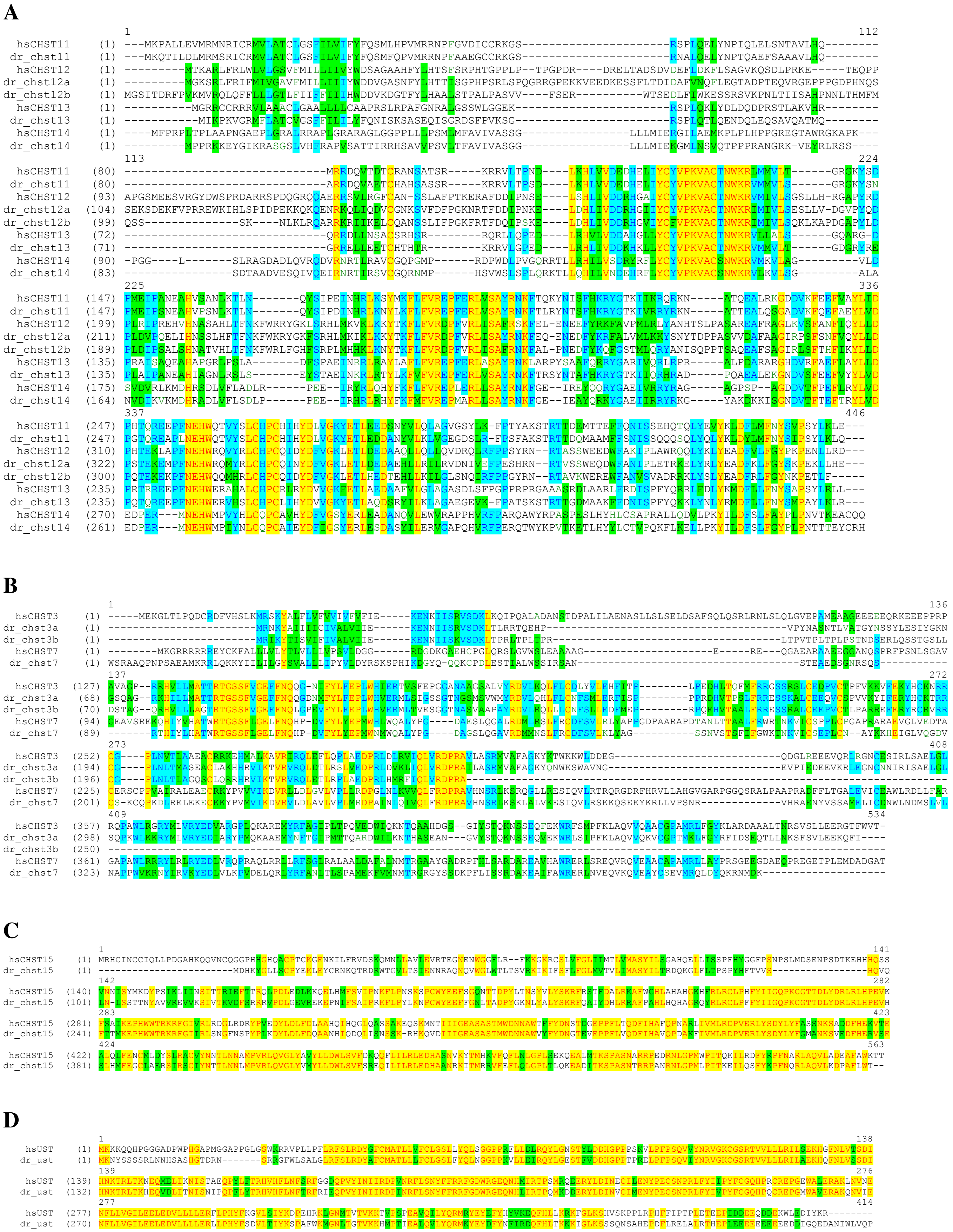
A
hsCHST11
dr_chst11
hsCHST12
dr_chst12a
dr_chst12b
hsCHST13
dr_chst13
hsCHST14
dr_chst14
B
hsCHST3
dr_chst3a
dr_chst3b
hsCHST7
dr_chst7
C
hsCHST15
dr_chst15
D
hsUST
dr_ust

E

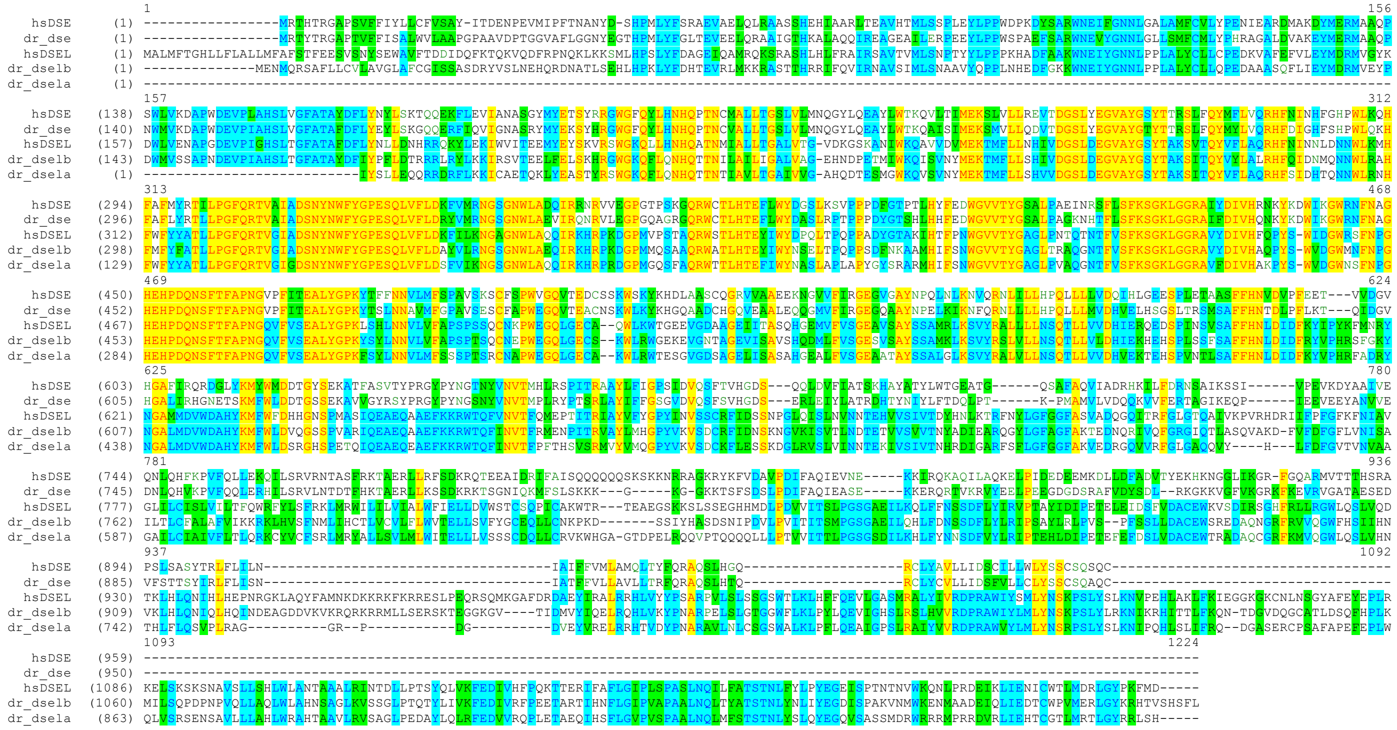

Supplement: S1 Fig — A, alignment of CS/DS 4-O sulfotransferases. B, CS/DS 6-O sulfotransferases. C, CS/DS 4-sulfate 6-O sulfotransferases. D, CS/DS 2-O-sulfotransferases and E, CS/DS epimerases. Identical amino acids are marked with red letters on yellow background, conserved residues are indicated by blue letters on light blue background and blocks of similar residues marked with black letters on green background. (PDF) [file pone.0121957.s001.pdf]
